# Large-scale recent expansion of European patrilineages shown by population resequencing

**DOI:** 10.1038/ncomms8152

**Published:** 2015-05-19

**Authors:** Chiara Batini, Pille Hallast, Daniel Zadik, Pierpaolo Maisano Delser, Andrea Benazzo, Silvia Ghirotto, Eduardo Arroyo-Pardo, Gianpiero L. Cavalleri, Peter de Knijff, Berit Myhre Dupuy, Heidi A. Eriksen, Turi E. King, Adolfo López de Munain, Ana M. López-Parra, Aphrodite Loutradis, Jelena Milasin, Andrea Novelletto, Horolma Pamjav, Antti Sajantila, Aslıhan Tolun, Bruce Winney, Mark A. Jobling

**Affiliations:** 1 Department of Genetics, University of Leicester, University Road, Leicester LE1 7RH, UK; 2 Department of Life Sciences and Biotechnology, University of Ferrara, Via Borsari 46, 44121 Ferrara, Italy; 3 Laboratory of Forensic and Population Genetics, Department of Toxicology and Health Legislation, Faculty of Medicine, Complutense University, E-28040 Madrid, Spain; 4 Molecular and Cellular Therapeutics, The Royal College of Surgeons in Ireland, 123 St Stephen's Green, Dublin 2, Ireland; 5 Department of Human Genetics, Leiden University Medical Centre, 2300 RC Leiden, The Netherlands; 6 Norwegian Institute of Public Health, Division of Forensic Sciences, N-0027 Oslo, Norway; 7 Centre of Arctic Medicine, Thule Institute, University of Oulu, PO Box 7300, 90014 Oulu, Finland; 8 Utsjoki Health Care Centre, 99980 Utsjoki, Finland; 9 Department of Neurosciences, University of the Basque Country, San Sebastián 20014, Spain; 10 Committee for Thalassemia of the Central Council of Health, Ministry of Health, 8 Merlin, Athens 106 71, Greece; 11 Institute of Human Genetics, School of Dental Medicine, University of Belgrade, 11000 Belgrade, Serbia; 12 Department of Biology, Tor Vergata University, Via della Ricerca Scientifica, 00133 Rome, Italy; 13 Network of Forensic Science Institutes, Institute of Forensic Medicine, , PO Box 31, 1363 Budapest, Hungary; 14 Department of Forensic Medicine, Hjelt Institute, University of Helsinki, PO Box 40, Helsinki FI-00014, Finland; 15 Institute of Applied Genetics, Department of Molecular and Medical Genetics, University of North Texas Health Science Center, 3500 Camp Bowie Boulevard, Fort Worth, Texas 76107, USA; 16 Department of Molecular Biology and Genetics, Boğaziçi University, Bebek, 34342 Istanbul, Turkey; 17 Department of Oncology, University of Oxford, Roosevelt Drive Oxford, Oxford OX3 7DQ, UK

## Abstract

The proportion of Europeans descending from Neolithic farmers ∼10 thousand years ago (KYA) or Palaeolithic hunter-gatherers has been much debated. The male-specific region of the Y chromosome (MSY) has been widely applied to this question, but unbiased estimates of diversity and time depth have been lacking. Here we show that European patrilineages underwent a recent continent-wide expansion. Resequencing of 3.7 Mb of MSY DNA in 334 males, comprising 17 European and Middle Eastern populations, defines a phylogeny containing 5,996 single-nucleotide polymorphisms. Dating indicates that three major lineages (I1, R1a and R1b), accounting for 64% of our sample, have very recent coalescent times, ranging between 3.5 and 7.3 KYA. A continuous swathe of 13/17 populations share similar histories featuring a demographic expansion starting ∼2.1–4.2 KYA. Our results are compatible with ancient MSY DNA data, and contrast with data on mitochondrial DNA, indicating a widespread male-specific phenomenon that focuses interest on the social structure of Bronze Age Europe.

## Abstract (web summary)

The origins and antiquity of the people of Europe has been much debated. Here, the authors sequence 3.7 Mb of the Y chromosome in over 300 Europeans and Middle Easterners and show a recent, continent-wide and male-specific expansion dating back to the Bronze Age.

## Graphical abstract

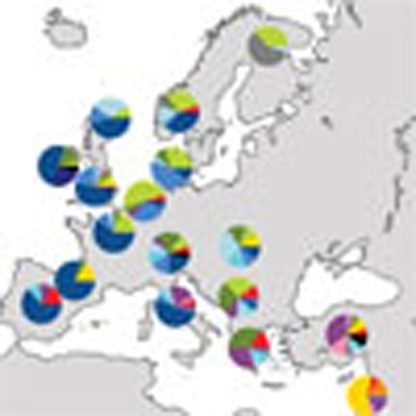

Controversy has surrounded the origins and antiquity of the people of Europe, focused on the proportions descending from Neolithic farmers originating ∼10 thousand years ago (KYA), or from earlier Palaeolithic hunter-gatherers. Early studies observed a European SE–NW cline in classical gene frequency data which was ascribed to demic diffusion of farmers^1^, or, in an alternative view, to the first Palaeolithic colonization^2^. More recent autosomal genome-wide SNP data sets reflect current population structure^3^,^4^ and admixture during the last 3,000 years^5^, but have provided little insight into older population processes.

Most debate on European prehistory has been stimulated by analyses of uniparentally-inherited markers. Spatial patterns in maternally-inherited mitochondrial DNA (mtDNA) are non-clinal, with age estimates of haplogroups (hg) taken to suggest a major Palaeolithic contribution^6^. Analyses of diversity in the male-specific region of the Y chromosome (MSY) show significant frequency clines in major lineages^7^, and geographical distributions and dates based on short-tandem repeats (STRs) have led to interpretations of both Palaeolithic^8^ and Neolithic^9^ major components. The most frequent western European lineage, hg R1b-M269, was originally believed to have originated in the Palaeolithic^10^, but in more recent analysis was assigned a Neolithic origin^11^, a claim challenged in turn^12^ on the basis of STR choice and sample ascertainment. In general, dates based on STRs are problematic because of uncertainty about appropriate mutation rates, and possible long-term mutation saturation due to their stepwise mutation processes^13^. Palaeolithic dates for the major lineages are challenged by scanty ancient MSY DNA data, which suggest a marked discontinuity between 5–7 KYA and the present^14^.

A major cause of the controversy about MSY evidence is that unbiased estimates of diversity and time depth have until recently been impossible to obtain in large samples. Next-generation sequencing (NGS) generally offers unbiased ascertainment of MSY SNPs, providing phylogenies in which topologies inform about past demography, and branch lengths are in principle proportional to time, avoiding dating problems associated with STRs. Some insights have emerged from recent work^15^,^16^, but no systematic population-based NGS study across Europe has yet been undertaken.

Here, we use targeted NGS of European and Middle Eastern populations to show that Europe was affected by a major continent-wide expansion in patrilineages that post-dates the Neolithic transition. Resequencing at high coverage of 3.7 Mb of MSY DNA, in each of 334 males comprising 17 population samples, defines an unbiased phylogeny containing 5,996 high-confidence single-nucleotide polymorphisms (SNPs). Dating indicates that three major lineages (I1, R1a and R1b), accounting for 64% of the sampled chromosomes, have very recent coalescent times, ranging between 3.5 and 7.3 KYA. In demographic reconstructions^17^ a continuous swathe of 13/17 populations from the Balkans to the British and Irish Isles share similar histories featuring a minimum effective population size ∼2.1–4.2 KYA, followed by expansion to the present. Together with other data on maternally inherited mtDNA^16^,^18^ and autosomal DNA^19^, our results indicate a recent widespread male-specific phenomenon that may point to social selection, and refocuses interest on the social and population structure of Bronze Age Europe.

## Results

### Samples and approach

To address the lack of a systematic population-based NGS study of European MSY diversity, we assembled a collection of 20 randomly chosen male DNA samples from each of 17 populations from Europe and the Middle East (Supplementary Table 1). We used a sequence-capture approach to successfully generate 3.7 Mb of unique MSY sequence from 334 of the total set of 340 males. Mean read-depth of 51 × allowed us to call a set of 5,996 high-confidence SNPs (Supplementary Data 1), with a minimum 6 × read-depth. SNP calls were validated using publicly available Y-chromosome sequence information from genome-wide data (Supplementary Table 2).

SNPs have been deposited in NCBI dbSNP database and ss numbers can be found in Supplementary Data 1.

### Phylogeography of European MSY lineages

**Figure 1:**
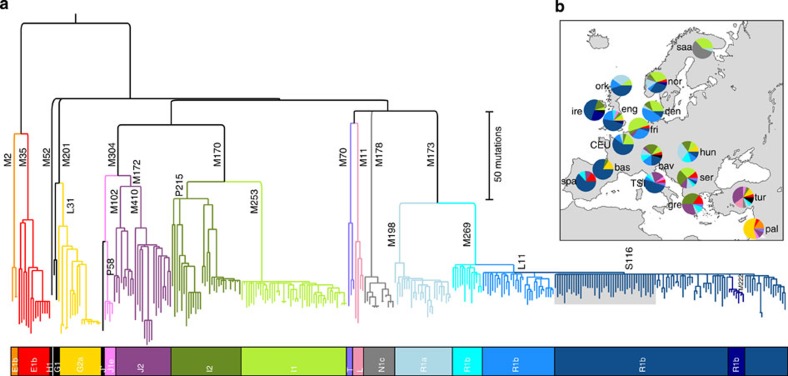
Phylogeny and geographical distribution of European MSY lineages. (**a**) Maximum-parsimony tree of European MSY lineages defined here by resequencing. Branch lengths are proportional to molecular divergence among haplotypes. Key mutation names are given next to some branches, and haplogroup names^20^ in the coloured bar below. Three sporadic haplogroups are coloured in black. The grey box within hg R1b-M269 shows the star phylogeny referred to in the text. (**b**) Map with pie-charts showing frequencies of Y-chromosome haplogroups (defined and coloured as in part **a**) in 17 populations from Europe and the Near East. Population abbreviations are as follows: bas: Basque; bav: Bavaria; CEU: Utah residents with Northern and Western European ancestry from the CEPH collection (France); den: Denmark; eng: England; fri: Frisia; gre: Greece; hun: Hungary; ire: Ireland; nor: Norway; ork: Orkney; pal: Palestinians; saa: Saami; ser: Serbia; spa: Spain; TSI: Toscani in Italia (Italy); tur: Turkey.

We constructed a maximum-parsimony tree displaying the phylogenetic relationships between SNP haplotypes (Fig. 1a; Supplementary Fig. 1), rooted by reference to two MSY sequences^13^ from the basal haplogroups A and B. Our sequenced regions cover many previously known SNPs, which allowed us to apply established haplogroup names^20^ to clades. Figure 1b shows the geographical distribution of these haplogroups in our samples, which is consistent with previous studies of specific SNPs using larger per-population sample sizes^10^. As expected, the commonest haplogroup is R1b-M269 (43.1%), with highest frequency in the north-west, followed by I1-M253 (13.8%), I2-P215 (9.0%), R1a-M198 (7.5%) and J2-M172 (7.5%). Some clades show geographically-restricted distributions, with hg N1c-M178 being most frequent in the Saami, and sub-lineages of haplogroups E, G and J prevalent in the Mediterranean area.

The shapes of different clades within the tree (Fig. 1a) vary greatly. Haplogroups E1b-M35, G2a-L31, I2-P215, J2-M172, L-M11 and T-M70 contain long branches with deep-rooting nodes, whereas I1-M253, N1c-M178, R1a-M198 and R1b-M269 show much shallower genealogies. Haplogroup R1b-M269 is particularly striking, containing a remarkable star phylogeny within which 44 terminal branches (13.2% of the total), found in 13 of the 17 sampled populations, descend as a multifurcation from a single node without any sub-structure whatsoever, despite the extensive nature of the sequencing carried out. These qualitative features of the phylogeny are supported by values of the average number of mutations from the ancestral node to branch tips, and also by estimates of time-to-most-recent-common-ancestor (TMRCA) (Table 1) derived by two different methods. Considering haplogroups R1b-M269, R1a-M198 and I1-M253, and the 95% highest posterior density intervals of their TMRCAs, 64% of the MSY sequences sampled in our study descend from three ancestors who each lived more recently than ∼7.3 KYA.

### Inferences on demographic history

Although it offers a tool to formulate hypotheses, the phylogeographic analysis above is limited in its power to illuminate demographic history. To further understand past demography, we applied a population approach: Table 2 shows diversity parameters for the 17 populations. When we consider the diversity from a molecular perspective (as the number of polymorphic sites) the highest diversity is in Turkey and Greece, closely followed by other southern populations (including Palestinians), and the lowest in Saami and Orkney. Consistent with this, there is a significant correlation of decreasing diversity both from south to north, and east to west (Supplementary Table 3), a clinal pattern that might be compatible with a model of demic diffusion from the Middle East. Considering instead the distribution of haplotypes, assessed as median number of singletons (here defined as variants that appear only once within a given population), by far the highest diversity is seen in Turkey and Greece, with the lowest diversity in the Saami and Palestinians—probably reflecting the effect of recent isolation and drift. Neither this measure, nor its standard deviation, shows any significant correlation with either latitude or longitude (Supplementary Table 3).

**Figure 2:**
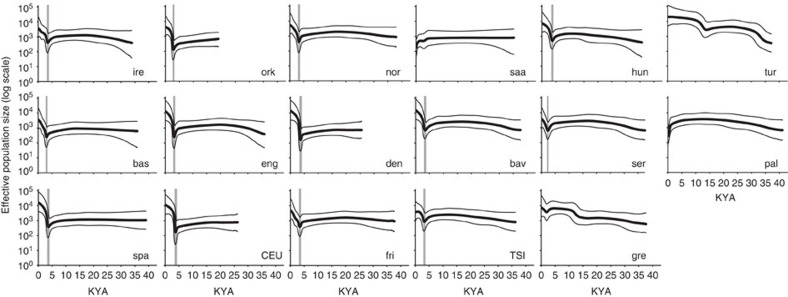
Bayesian skyline plots. Thick black lines indicate the median for effective population size (*N*_e_) and thinner grey lines show 95% higher posterior density intervals. Grey shading indicates the confidence intervals of the time estimate of the minimum effective population size before the expansion, based on the limits of the 95% CI of the mutation rate. Population abbreviations are as follows: bas: Basque; bav: Bavaria; CEU: Utah residents with Northern and Western European ancestry from the CEPH collection (France); den: Denmark; eng: England; fri: Frisia; gre: Greece; hun: Hungary; ire: Ireland; nor: Norway; ork: Orkney; pal: Palestinians; saa: Saami; ser: Serbia; spa: Spain; TSI: Toscani in Italia (Italy); tur: Turkey.

Bayesian skyline plots (BSPs) (Fig. 2) reveal the variation of effective population size with time^21^. The plots are consistent with patterns seen in the relative numbers of singletons, described above, in that the Saami and Palestinians show markedly different demographic histories compared with the rest, featuring very recent reductions, while the Turks and Greeks show evidence of general expansion, with increased growth rate around 14 KYA. A different pattern is seen in the remaining majority (13/17) of populations, which share remarkably similar histories featuring a minimum effective population size ∼2.1–4.2 KYA (considering the 95% confidence intervals (CIs) reported in Supplementary Table 4), followed by expansion to the present. Considering only these 13 populations, the only significant geographical correlation is of decreasing diversity in the number of polymorphic sites from east to west (Supplementary Table 3); notably, there is no significant correlation between the age at which effective population size was at a minimum before expansion (Supplementary Table 4), and either latitude or longitude (Supplementary Table 3). Taken together, the very recent age of the demographic shift and its lack of geographical pattern suggest that its origin is distinct from that of the diffusion of agriculture.

In contrast to BSPs, approximate Bayesian computation (ABC) offers an alternative model-based approach to understanding complex population histories, based on coalescent simulations^22^,^23^. We analysed several demographic models including single population expansion, reduction or combinations of the two (see Supplementary Fig. 2). Despite the high-molecular resolution of our data, the fact that they originate from a single locus did not allow precise estimation of demographic parameters, but the ABC generally confirmed the population size dynamics reconstructed by the BSP analysis (with some exceptions: Supplementary Tables 5–7). The proportion of the parameter variance explained by the summary statistics (*R*^2^) is in most cases higher than 10% (and hence generally considered as a good estimation), but the 95% credible intervals are wide. This is particularly true for T1, the time of the start of the demographic change (reduction or expansion), thus preventing us from drawing any conclusion about the timing of these events (whether post Neolithic, or more ancient) from the ABC analysis.

In general, the non-parametric genealogical approach represented by BSPs better explores the variation found in our data, compared with a more conservative ABC analysis based on a single locus. We note that both analyses assume panmixia, and that population structure might influence effective population size estimates. However, it seems improbable that such an effect would extend to so many populations.

## Discussion

Our approach has led to the confident identification of many MSY sequence variants in European population samples and a highly resolved phylogeny, but our conclusions are also influenced by a more contentious factor, the choice of mutation rate. We chose a rate (1.0 (0.92–1.09) × 10^−9^ per bp per year, considering a 30-year generation time) based on the observation of 609 MSY mutations (excluding palindromic regions) in Icelandic deep-rooting pedigrees^24^. The point estimate of this rate is the same as an earlier pedigree-based estimate in which only four mutations were observed^25^, and which we applied in our broader MSY-phylogeny study^13^.

We note that the rate we have used is higher than the estimate of 0.76 (0.67–0.86) × 10^−9^ per bp per year based on counting the ‘missing’ mutations in the genome of the Ust’-Ishim male^26^, radiocarbon dated to ∼45,000 YBP. Other studies^27^,^28^ have inferred slower mutation rates (0.62 or 0.64 × 10^−9^) based on scaling the genome-wide *de novo* rate to account for male-specific transmission, though this has been criticized^29^, and is not consistent with phylogenetic mutation rates estimated from human–chimpanzee MSY comparisons^30^,^31^. Some have chosen to calibrate the pedigree mutation rate against external (for example, archaeological) data^15^,^32^, but we have rejected this idea, firstly because of uncertainty over how archaeological date estimates correlate with demographic changes, and secondly because we have used a coalescent-based dating method that itself models genealogies. Despite recent advances, mutation rate remains a difficult issue, and more data are needed.

**Figure 3:**
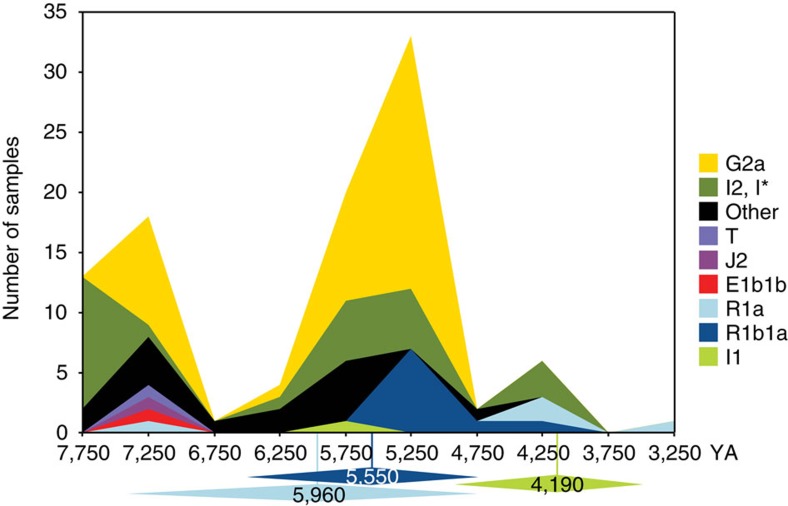
Timeline of MSY ancient DNA data. The graph shows stacked frequencies of MSY haplogroups in ancient European DNA samples, based on data from 98 individuals, and binned into 500-year intervals. ‘other’ includes C1, F*, H2, R*, R1, R1b(xR1b1a). Below the timeline are indicated BEAST point estimates and highest posterior density intervals for three relevant haplogroups.

The recent and rapid continent-wide demographic changes we observe suggest a remarkably widespread transition affecting paternal lineages. This picture is confirmed in an independent analysis of MSY diversity in the pooled HGDP CEPH panel European samples^16^, and is compatible with current (*n*=98) ancient DNA data for MSY (Fig. 3; Supplementary Table 8), in which hgs R1a, R1b and I1 are absent or rare in sites dating before 5 KYA, whereas hgs G2a and I2 are prevalent.

Analyses of ancient autosomal sequence data^19^ demonstrate discontinuity 7–5 KYA between western European hunter-gatherers, tending to have genetic affinity to northern Europeans, and farmers, resembling southern Europeans. Consideration of the genomic ancestry of modern Europeans^33^ reveals ancestry from these two groups, but also from north Eurasian hunter-gatherers^33^. Recent analysis^34^ better defines this latter component, supporting a two-migration model into a hunter-gatherer substrate, involving an early-Neolithic (7–8 KYA) arrival of farming populations from the Near East, followed by a late-Neolithic (4.5 KYA) migration of pastoralists from the steppe region north of the Caspian Sea, whose genomic contribution is ubiquitous in modern Europeans. Ancient MSY sequences^34^ show that hgs R1a and R1b are present in the steppe much earlier than observed in any European sites (Supplementary Table 8), making this region a likely source for these MSY expansion lineages.

Ancient mtDNA data^18^ also indicate large-scale population discontinuity since the Neolithic transition, with a massive shift in haplogroup composition ∼7.5 KYA between Central European hunter-gatherers (carrying exclusively hg U lineages) and farmers (a much broader range of hgs), followed by later fluctuations. Demographic inference from whole mtDNA sequences^16^, however, does not show recent and sudden expansion. This suggests that the recent events responsible for shaping modern MSY variation were male specific.

The period 4–5 KYA (the Early Bronze Age) is characterized by rapid and widespread change, involving changes in burial practices that might signify an emphasis on individuals or kin groups, the spread of horse riding, and the emergence of elites and developments in weaponry^35^. In principle male-driven social selection^36^ associated with these changes could have led to rapid local increases in the frequencies of introgressing haplogroups^34^, and subsequent spread, as has been suggested for Asia^37^. However, cultures across Europe remain diverse during this period; clarifying the temporal and geographical pattern of the shift will rely heavily on additional ancient DNA data.

## Methods

### Samples

DNA donors were recruited with informed consent (University of Leicester Research Ethics Committee reference: maj4-cb66). DNA was extracted from various sources including lymphoblastoid cell lines, peripheral blood and saliva. Samples (340) were included in the design from 17 populations (20 males each) across Europe and the Near East. Samples from Greece, Serbia, Hungary, Germany (Bavaria), Spanish Basque country, central Spain, Netherlands (Frisia), Denmark, Norway, Finland (Saami), England^38^ (Herefordshire and Worcestershire), Orkney^38^, Ireland and Turkey were collected by the authors. Twenty random Palestinian male samples were purchased from the National Laboratory for the Genetics of Israeli Populations ( www.tau.ac.il/medicine/NLGIP). Finally, samples from two HapMap^39^ populations were used, both to supplement the population data set and to provide data on externally analysed samples for validation purposes: the Centre d'Etude du Polymorphisme Humain (CEPH) collection in Utah, USA, with ancestry from Northern and Western Europe (CEU) and the Toscani in Italia (TSI). After the initial analyses, one English and one Spanish individual were identified as females and therefore removed from all downstream analyses in this study, reducing the final number of samples to 338. For further details on samples see Supplementary Table 1.

### Bait design for target enrichment

For target enrichment Agilent SureSelect (Agilent Technologies Inc., CA, USA) hybridization capture was used. RNA baits were designed using Agilent eArray with default parameters for Illumina Paired-End Long Read sequencing (bait length: 120 bp; design strategy: centred; tiling frequency: 1 × ; avoid overlap: 20 bp; strand: sense; avoid regions: repeat masker) and human reference sequence hg19/GRCh37 (February 2009). Boosting was used for ‘orphan’ (located >20 bp from flanking baits) and GC-rich (⩾63%) baits by direct replication (orphans 2 × , GC-rich 3 × ).

In this study we focus on the eight X-degenerate regions^31^ of the Y chromosome which are likely to yield interpretable sequence data; other captured regions are discussed elsewhere^13^. The total length of targeted regions was ∼8.6 Mb, and following capture design and the necessary repeat masking, the designed baits covered 2.3 Mb. Coordinates of the eight targeted regions can be found in Supplementary Data 2.

### Sequencing and data processing

Genomic DNA (3–5 μg) was used for library preparation and target enrichment using Agilent SureSelect^XT^ Target Enrichment System for Illumina Paired-End Sequencing Library kit (version 1.3). In order to obtain larger insert sizes, DNA samples were fragmented to ∼250–600 bp without size selection. This resulted in a mean insert size of 330 bp, which increases recovery of sequence data from bait-adjacent regions. Sequencing was done on an Illumina HiSeq 2000 instrument (Illumina, CA, USA) with paired-end 100-bp run to high coverage. Library preparation, target enrichment and sequencing were carried out at the High-Throughput Genomics Centre at the Wellcome Trust Centre for Human Genetics, University of Oxford, UK.

Base calling was done using Illumina Bustard^40^ and quality control with FastQC^41^. Sequence data were mapped to the human genome reference (GRCh37) using Stampy v1.0.20 (ref. 42). Local realignment was done using The Genome Analysis Toolkit (GATK) v2.6-5 (ref. 43), followed by duplicate read marking with Picard v1.86 (ref. 44) and base quality score recalibration also with GATK. The individual steps and parameters used are listed in Supplementary Table 9.

### Variant calling and filtering

Owing to larger insert sizes (see above) and high efficiency of sequence capture, high sequence coverage was obtained not only at baited regions but also at ∼300 bp flanking the enrichment baits. Therefore, the original bait coordinates were modified by adding 300 bp to either side of each bait followed by merging the overlapping coordinates and increasing the size of the analysed region to 4,433,580 bp.

Data on the 338 male samples described above were co-analysed with simultaneously generated data on an additional 117 samples, described elsewhere^13^. Variant calling was done using the samtools mpileup v0.1.19 multi-sample option, calling all samples simultaneously (Supplementary Table 9). In total 19,276 raw SNPs were called from 455 male samples.

Raw variants were filtered using vcftools v0.1.11 (ref. 45) and in-house Perl scripts. Filters used for the final data are listed in Supplementary Table 9. As well as the two females, seven samples (four from the European population set) were removed from the final data set due to missing >5% of calls. The filtered data set included 13,474 variant sites from 448 samples, from 0 to 643 missing calls per individual, with an average call rate of 99.8%.

To recover as many missing genotypes as possible for subsequent analyses, they were divided into three groups based on read-depth: DP 0—the genotype call was discarded; DP 2–6—the raw call was accepted; all other cases—the sites were re-called using a single-sample approach to obtain the DP4 field in the vcf, the bam file was checked manually, and the most probable allele was inferred by comparing the bam file with the information contained in the DP4 field. After this procedure, 213/13,474 sites still lacked genotype calls, leading to a final number of 13,261 sites for further analyses.

Having applied the above filters to variant sites, it was necessary to apply the same criteria to non-variant sites. We calculated the depth per sample per site using the GATK DepthOfCoverage tool, filtered for base quality 20 and mapping quality 50, and then applied the criterion of ⩾6 × coverage in ⩾95% of samples. This led to a reduction in the figure of base pairs sequenced from 4,433,580 to 3,724,156 bp. The corresponding coordinates (Supplementary Data 2) were used for all downstream analysis.

Mean raw sequence coverage per sample across the 3,724,156 bp of analysed regions was calculated using Picard v1.93. Sequence depth for the 448 samples varied from 25 × to 85 × per sample, with the average of 51 × . Supplementary Table 1 shows sequence depth information for the 338 male samples described here. The final European data set used here, excluding cases with >5% missing calls, includes 334 individuals and 5,996 SNPs (Supplementary Data 3).

### Validation

*In silico* validation of the 13,261 filtered SNP calls was done using two previously published data sets: genomes sequenced to high-coverage with self-assembling DNA nanoarrays by Complete Genomics^46^, and Omni2.5 BeadChip genotype data produced at the Broad Institute as part of the 1,000 Genomes Project^47^. Our samples included 4 and 39 HapMap individuals overlapping with these two data sets, respectively. A Perl script was written to compare the SNP calls in our variant set to overlapping samples and positions in the control sets.

Of the 888 variant sites shared between our data and the Complete Genomics data across four overlapping samples, the false positive and false negative error rates were both 0%. When compared with the Omni data across 241 variant sites and 39 overlapping samples, the error rates were 0.13% for false positives and 1.82% for false negatives. However, all the false calls originated from only 19 variant sites.

To shed light on these comparatively high error rates, we also compared Complete Genomics and Omni data for regions corresponding to our final analysed regions. Across 263 variant sites and 49 overlapping samples, we obtained false positive and false negative rates of 2.85 and 2.36%, respectively. The false calls originated from 30 sites and 15 of those overlap with the sites producing high error rates when comparing our data with Omni. Since the Complete Genomics data set is generally considered to have very high quality then this seems to indicate problems in making correct calls from Omni genotyping data. More detail is provided in Supplementary Table 2.

### Phylogenetic inference

PHYLIP v3.69 was used to create a maximum parsimony phylogenetic tree^48^. Three independent trees were constructed with dnapars using randomization of input order (seeds: 4941985, 62529981 and 38185313), each 10 times. Output trees of these runs were used to build a consensus tree with the consense programme included in PHYLIP package.

The tree was rooted using two Y-chromosomes belonging to haplogroups A and B which were sequenced in the complete data set^13^. FigTree v1.4.0^49^ was used to visualize the tree ( tree.bio.ed.ac.uk/software/figtree/).

### Haplogroup prediction

The presence of known markers was checked using AMY-tree v1.2 (refs 50, 51). This software was developed to predict MSY haplogroups from whole-genome sequence data using a list of known markers. Since our data do not cover the whole MSY but only a proportion of it, the software lacks sufficient information for haplogroup prediction. However, it can be used to deduce the presence and allelic states of known MSY markers present in sequence data. The AMY-tree v1.2 conversion file contains a list of 1,453 known Y-SNPs, of which 490 are present in our data. These 490 sites were used to assign a standard haplogroup to all our samples according to the Y Chromosome Consortium phylogenetic tree^20^ and its subsequent updates (Supplementary Table 1).

### TMRCA and ages of nodes

The TMRCA of the tree and of nodes of interest were estimated via two approaches:

- BEAST v1.8 (refs 17, 52): Markov chain Monte Carlo (MCMC) samples were based on 25,000,000 generations, logging every 1,000 steps, with the first 2,500,000 generations discarded as burn-in. Three runs were combined for analysis using LogCombiner. We used an exponential growth coalescent tree prior (growth rate prior: uniform(0–0.002)), HKY substitution model, and a strict clock with a substitution rate of 1.0 (95% CI: 0.92–1.09) × 10^−9^ mutations/nucleotide/year^24^. TMRCAs were estimated in a single run including all 17 European populations and assigning samples to specific clades in agreement with the MP tree shown in Fig. 1.
- Rho: A Perl script was written to calculate TMRCA and its standard deviation for any given clade within a PHYLIP outfile, using the rho statistic^53^,^54^. A scaled mutation rate of 268.5 (246.3–291.9) years per mutation was used, based on a published rate of 1.0 (95% CI: 0.92–1.09) × 10^−9^ mutations/nucleotide/year^24^ and the number of nucleotides in our regions of interest (3,724,156).

### Bayesian skyline plots

Bayesian skyline plots were generated using BEAST v1.8 (refs 17, 52). MCMC samples were based on 100,000,000 generations, logging every 1,000 steps, with the first 10,000,000 generations discarded as burn-in. We used a piecewise linear skyline model with 10 groups, a HKY substitution model, and a strict clock with a mean substitution rate of 1.0 × 10^−9^ mutations/nucleotide/year^24^ and a generation time of 30 years, consistent with 55. For the 13 populations showing a recent expansion in the BSP, the limits of the 95% CI of mutation rate^24^ (0.92–1.09 × 10^−9^) were used to define the CI of the time estimate of the minimum effective population size before the expansion (grey shading in Fig. 2).

### Intrapopulation diversity and geographical correlation

The number of polymorphic sites per population, Tajima’s *D*^56^, and Fu’s FS^57^ were calculated using Arlequin 3.5 (ref. 58). The number of singletons was calculated using Vcftools v0.1.11. Correlation tests between measures of genetic diversity and latitude and longitude were run in R^59^ with the function cor.test of the package stats.

### Approximate Bayesian computation

We generated one million simulated datasets for each tested model (Supplementary Fig. 2) with the programme FastSimcoal2 (ref. 60), simulating a single haploid locus of 3,724,156 bp. We summarized the data by means of the derived site frequency spectrum (-s -d flags in the command line) considering only categories with at least one observed polymorphic site. Ancestral states in the observed data were defined elsewhere^13^ using a custom script.

To compare models we applied the Logistic Regression procedure^61^. Model parameters were estimated by a locally weighted multivariate regression^22^ after a *logtan* transformation^62^ of the 10,000 best-fitting simulations from a specific model. To calculate the posterior probabilities for models and parameters we used R scripts from http://code.google.com/p/popabc/source/browse/#svn%2Ftrunk%2Fscripts, modified by AB and SG.

We also estimated the power of our ABC procedure to correctly recognize the true model calculating for each model the proportion of true positives and false positives. We evaluated 1,000 pseudo-observed data sets generated under each model, counting the number of times a specific model is correctly identified by the ABC procedure (true positives), and the number of times the same model is incorrectly selected as the true model (false positives).

### Demographic models and priors

We considered five models depicting different demographic histories, testing each model separately for each population (Supplementary Fig. 2). M1 is the simplest, in which the effective population size remains constant over time (uniform prior: 20–20,000). In M2 an ancient constant-sized population (uniform prior: 1,001–20,000) starts an exponential reduction T1 generations ago. The reduction spans LEX generations (uniform prior: 5–634), then the population returns to constant size (uniform prior: 20–1,000) T2 generations ago (uniform prior: 0–30). In M3 the reduction is followed by an expansion that starts T2 generations ago (with the effective population size, NER, drawn from an uniform prior: 20–1,000) until the present (uniform prior for the current effective population size, NC: 1,001–20,000). M4 is parameterized in the same way as M2, with an expansion instead of a reduction (NA uniform prior: 20–1,000; NC uniform prior: 1,001–20,000). In M5, the expansion ends at T2 followed by a reduction until present time (NEE uniform prior: 1,001–20,000; NC uniform prior: 20–1,000). We considered a generation time of 30 years. In all the models the Last Glacial Maximum (∼20,000 years ago) represents the upper bound of the time for the first demographic change (T1).

In each simulation the per-generation, per-site mutation rate^24^ is drawn from a normal distribution with mean 3.01 × 10^−8^ and 95% confidence intervals 2.77–3.26 × 10^−8^. DNA sequences were generated under a finite sites mutational model with no transition/transversion bias.

Perl scripts used in the analysis are available upon request.

## Additional information

**How to cite this article:** Batini, C. *et al*. Large-scale recent expansion of European patrilineages shown by population resequencing. *Nat. Commun.* 6:7152 doi: 10.1038/ncomms8152 (2015).

## Supplementary Material

Supplementary Information Supplementary Figures 1-2, Supplementary Tables 1-9 and Supplementary References

Supplementary Data 1 List of variants and correspondence with other studies. The data include ss (submitted SNP) numbers allowing variant information to be retrieved from dbSNP.

Supplementary Data 2 Target and sequenced regions coordinates.

Supplementary Data 3 Vcf file containing all samples and sites analysed in the study.

## Figures and Tables

**Table 1:** TMRCAs of major haplogroups in Europe estimated using two methods.

**hg**	***N***	**Mean mutations to root of hg**	**BEAST**		**rho**	
			**TMRCA/YA**	**95% HPD interval**	**TMRCA/YA**	**TMRCA range based on mutation rate CI/YA**
E1b-M35	13	65	17,800	15,400–20,500	17,450	16,010–18,970
G2a-L31	18	61	15,300	13,200–17,500	15,740	14,440–17,110
I1-M253	46	13	4,190	3,470–5,070	3,460	3,180–3,760
I2-P215	30	64	18,000	15,800–20,200	17,090	15,670–18,570
J2-M172	23	80	21,500	19,000–24,100	21,400	19,630–23,260
J2a-M410	15	58	15,200	13,200–17,300	15,700	14,400–17,070
J2b-M102	8	42	12,200	10,200–14,300	11,240	10,310–12,220
N1c-M178	13	18	3,600	2,640–4,670	4,770	4,380–5,190
R1a-M198	25	24	5,960	4,750–7,340	6,340	5,810–6,890
R1b-M269	144	18	5,550	4,750–6,500	4,890	4,480–5,310
R1b-L11	131	13	4,510	3,920–5,160	3,590	3,290–3,900
R1b-S116	100	12	4,210	3,700–4,790	3,320	3,050–3,610

hg: haplogroup; *N*: number of sequences; HPD: highest posterior density; TMRCA, time-to-most-recent-common-ancestor; YA: years ago.

**Table 2:** Diversity parameters for the 17 populations.

**pop**	***N***	***S***	**Median no. singletons** *	**s.d. singletons** *	***D***	***D P*** **-value**	**FS**	**FS** ***P*****-value**
bas	19	572	7	26.8	−1.318	0.061	−1.172	0.176
bav	20	1,089	14	59.4	−1.460	0.053	−0.413	0.271
CEU	20	618	13	20.5	−1.043	0.160	−1.098	0.171
den	20	609	12.5	10.9	−0.326	0.421	−0.775	0.221
eng	19	756	12	41.1	***−1.987***	***0.012***	−1.140	0.200
fri	20	825	8.5	56.4	−0.961	0.175	−0.579	0.212
gre	20	1,279	38.5	30.3	−1.361	0.061	−0.130	0.278
hun	20	784	12.5	13.0	−0.847	0.196	−0.604	0.240
ire	20	673	9	31.2	−1.479	0.051	−1.213	0.151
nor	20	902	9.5	57.5	−1.068	0.137	−0.494	0.224
ork	20	469	8.5	4.6	−0.996	0.158	−1.590	0.133
pal	19	1,034	2	48.4	−0.943	0.159	3.451	0.912
saa	19	455	1	27.4	0.795	0.846	4.127	0.947
ser	19	972	8	29.4	−0.865	0.187	−0.115	0.292
spa	19	828	15	15.2	−1.273	0.088	−0.535	0.239
TSI	20	1,026	13	57.9	***−1.678***	***0.034***	0.986	0.602
tur	20	1,745	52.5	55.9	***−2.058***	***0.008***	−0.091	0.310

*D*: Tajima’s *D* (bold italic values are significant); FS: Fu’s FS; *N*: number of individuals; *S*: number of polymorphic sites.

For population key, see Fig. 1 legend.

^*^Within population.

## References

[b1] Ammerman A. J. & Cavalli-Sforza L. L. Neolithic transition and the genetics of populations in Europe Princeton University Press (1984).

[b2] Richards M. . Paleolithic and neolithic lineages in the European mitochondrial gene pool. Am. J. Hum. Genet. 59, 185–203 (1996). 8659525 PMC1915109

[b3] Novembre J. . Genes mirror geography within Europe. Nature 456, 98–101 (2008). 18758442 10.1038/nature07331 PMC2735096

[b4] Lao O. . Correlation between genetic and geographic structure in Europe. Curr. Biol. 18, 1241–1248 (2008). 18691889 10.1016/j.cub.2008.07.049

[b5] Hellenthal G. . A genetic atlas of human admixture history. Science 343, 747–751 (2014). 24531965 10.1126/science.1243518 PMC4209567

[b6] Richards M. . Tracing European founder lineages in the near eastern mtDNA pool. Am. J. Hum. Genet. 67, 1251–1276 (2000). 11032788 PMC1288566

[b7] Rosser Z. H. . Y-chromosomal diversity within Europe is clinal and influenced primarily by geography, rather than by language. Am. J. Hum. Genet. 67, 1526–1543 (2000). 11078479 10.1086/316890 PMC1287948

[b8] Rootsi S. . Phylogeography of Y-chromosome haplogroup I reveals distinct domains of prehistoric gene flow in Europe. Am. J. Hum. Genet. 75, 128–137 (2004). 15162323 10.1086/422196 PMC1181996

[b9] Semino O. . Origin, diffusion, and differentiation of Y-chromosome haplogroups E and J: inferences on the neolithization of Europe and later migratory events in the Mediterranean area. Am. J. Hum. Genet. 74, 1023–1034 (2004). 15069642 10.1086/386295 PMC1181965

[b10] Semino O. . The genetic legacy of Paleolithic Homo sapiens sapiens in extant Europeans: a Y chromosome perspective. Science 290, 1155–1159 (2000). 11073453 10.1126/science.290.5494.1155

[b11] Balaresque P. . A predominantly Neolithic origin for European paternal lineages. PLoS Biol. 8, e1000285 (2010). 20087410 10.1371/journal.pbio.1000285 PMC2799514

[b12] Busby G. B. . The peopling of Europe and the cautionary tale of Y chromosome lineage R-M269. Proc. Biol. Sci. 279, 884–892 (2012). 21865258 10.1098/rspb.2011.1044 PMC3259916

[b13] Hallast P. . The Y-chromosome tree bursts into leaf: 13,000 high-confidence SNPs covering the majority of known clades. Mol. Biol. Evol. 32, 661–673 (2015). 25468874 10.1093/molbev/msu327 PMC4327154

[b14] Veeramah K. R. & Hammer M. F. The impact of whole-genome sequencing on the reconstruction of human population history. Nat. Rev. Genet. 15, 149–162 (2014). 24492235 10.1038/nrg3625

[b15] Francalacci P. . Low-pass DNA sequencing of 1200 Sardinians reconstructs European Y-chromosome phylogeny. Science 341, 565–569 (2013). 23908240 10.1126/science.1237947 PMC5500864

[b16] Lippold S. . Human paternal and maternal demographic histories: insights from high-resolution Y chromosome and mtDNA sequences. Investig. Genet. 5, 13 (2014). 10.1186/2041-2223-5-13 PMC4174254 25254093

[b17] Drummond A. J. & Rambaut A. BEAST: Bayesian evolutionary analysis by sampling trees. BMC Evol. Biol. 7, 214 (2007). 17996036 10.1186/1471-2148-7-214 PMC2247476

[b18] Brandt G. . Ancient DNA reveals key stages in the formation of central European mitochondrial genetic diversity. Science 342, 257–261 (2013). 24115443 10.1126/science.1241844 PMC4039305

[b19] Olalde I. . Derived immune and ancestral pigmentation alleles in a 7,000-year-old Mesolithic European. Nature 507, 225–228 (2014). 24463515 10.1038/nature12960 PMC4269527

[b20] Karafet T. M. . New binary polymorphisms reshape and increase resolution of the human Y-chromosomal haplogroup tree. Genome Res. 18, 830–838 (2008). 18385274 10.1101/gr.7172008 PMC2336805

[b21] Ho S. Y. & Shapiro B. Skyline-plot methods for estimating demographic history from nucleotide sequences. Mol. Ecol. Resour. 11, 423–434 (2011). 21481200 10.1111/j.1755-0998.2011.02988.x

[b22] Beaumont M. A., Zhang W. & Balding D. J. Approximate Bayesian computation in population genetics. Genetics 162, 2025–2035 (2002). 12524368 10.1093/genetics/162.4.2025 PMC1462356

[b23] Bertorelle G., Benazzo A. & Mona S. ABC as a flexible framework to estimate demography over space and time: some cons, many pros. Mol. Ecol. 19, 2609–2625 (2010). 20561199 10.1111/j.1365-294X.2010.04690.x

[b24] Helgason A. . The Y-chromosome point mutation rate in humans. Nat. Genet. doi:; DOI: 10.1038/ng.3171 (2015). 25807285

[b25] Xue Y. . Human Y chromosome base-substitution mutation rate measured by direct sequencing in a deep-rooting pedigree. Curr. Biol. 19, 1453–1457 (2009). 19716302 10.1016/j.cub.2009.07.032 PMC2748900

[b26] Fu Q. . Genome sequence of a 45,000-year-old modern human from western Siberia. Nature 514, 445–449 (2014). 25341783 10.1038/nature13810 PMC4753769

[b27] Mendez F. L. . An African American paternal lineage adds an extremely ancient root to the human Y chromosome phylogenetic tree. Am. J. Hum. Genet. 92, 454–459 (2013). 23453668 10.1016/j.ajhg.2013.02.002 PMC3591855

[b28] Scozzari R. . An unbiased resource of novel SNP markers provides a new chronology for the human Y chromosome and reveals a deep phylogenetic structure in Africa. Genome Res. 24, 535–544 (2014). 24395829 10.1101/gr.160788.113 PMC3941117

[b29] Elhaik E., Tatarinova T. V., Klyosov A. A. & Graur D. The 'extremely ancient' chromosome that isn't: a forensic bioinformatic investigation of Albert Perry's X-degenerate portion of the Y chromosome. Eur. J. Hum. Genet. 22, 1111–1116 (2014). 24448544 10.1038/ejhg.2013.303 PMC4135414

[b30] Kuroki Y. . Comparative analysis of chimpanzee and human Y chromosomes unveils complex evolutionary pathway. Nat. Genet. 38, 158–167 (2006). 16388311 10.1038/ng1729

[b31] Skaletsky H. . The male-specific region of the human Y chromosome: a mosaic of discrete sequence classes. Nature 423, 825–837 (2003). 12815422 10.1038/nature01722

[b32] Poznik G. D. . Sequencing Y chromosomes resolves discrepancy in time to common ancestor of males versus females. Science 341, 562–565 (2013). 23908239 10.1126/science.1237619 PMC4032117

[b33] Lazaridis I. . Ancient human genomes suggest three ancestral populations for present-day Europeans. Nature 513, 409–413 (2014). 25230663 10.1038/nature13673 PMC4170574

[b34] Haak W. . Massive migration from the steppe was a source for Indo-European languages in Europe. Nature doi:10.1038/nature14317 (2015). 10.1038/nature14317 PMC5048219 25731166

[b35] Cunliffe B. Europe Between the Oceans: 9000 BC-AD 1000 Yale University Press (2011).

[b36] Zerjal T. . The genetic legacy of the Mongols. Am. J. Hum. Genet. 72, 717–721 (2003). 12592608 10.1086/367774 PMC1180246

[b37] Balaresque P. . Y-chromosome descent clusters and male differential reproductive success: young lineage expansions dominate Asian pastoral nomadic populations. Eur. J. Hum. Genet. doi:; DOI: 10.1038/ejhg.2014.285 (2015). PMC4430317 25585703

[b38] Winney B. . People of the British Isles: preliminary analysis of genotypes and surnames in a UK-control population. Eur. J. Hum. Genet. 20, 203–210 (2012). 21829225 10.1038/ejhg.2011.127 PMC3260910

[b39] International HapMap Consortium. Integrating common and rare genetic variation in diverse human populations. Nature 467, 52–58 (2010). 20811451 10.1038/nature09298 PMC3173859

[b40] Kao W. C., Stevens K. & Song Y. S. BayesCall: a model-based base-calling algorithm for high-throughput short-read sequencing. Genome Res. 19, 1884–1895 (2009). 19661376 10.1101/gr.095299.109 PMC2765266

[b41] Andrews S. FastQC http://www.bioinformatics.babraham.ac.uk/projects/fastqc/ (2012).

[b42] Lunter G. & Goodson M. Stampy: a statistical algorithm for sensitive and fast mapping of Illumina sequence reads. Genome Res. 21, 936–939 (2011). 20980556 10.1101/gr.111120.110 PMC3106326

[b43] DePristo M. A. . A framework for variation discovery and genotyping using next-generation DNA sequencing data. Nat. Genet. 43, 491–498 (2011). 21478889 10.1038/ng.806 PMC3083463

[b44] Wysoker A., Tibbetts K. & Fennell T. Picard v1.86, available from http://picard.sourceforge.net/ (2009).

[b45] Danecek P. . The variant call format and VCFtools. Bioinformatics 27, 2156–2158 (2011). 21653522 10.1093/bioinformatics/btr330 PMC3137218

[b46] Drmanac R. . Human genome sequencing using unchained base reads on self-assembling DNA nanoarrays. Science 327, 78–81 (2010). 19892942 10.1126/science.1181498

[b47] 1000 Genomes Project Consortium. An integrated map of genetic variation from 1,092 human genomes. Nature 491, 56–65 (2012). 23128226 10.1038/nature11632 PMC3498066

[b48] Felsenstein J. PHYLIP (Phylogeny Inference Package) version 3.6. Department of Genome Sciences, University of Washington, (2005).

[b49] Rambaut A. Fig.Tree. Tree Figure Drawing Tool, version 1.4.0. available at http://tree.bio.ed.ac.uk/software/figtree/ (2006–2012).

[b50] Van Geystelen A., Decorte R. & Larmuseau M. H. AMY-tree: an algorithm to use whole genome SNP calling for Y chromosomal phylogenetic applications. BMC Genomics 14, 101 (2013). 23405914 10.1186/1471-2164-14-101 PMC3583733

[b51] Van Geystelen A., Decorte R. & Larmuseau M. H. Updating the Y-chromosomal phylogenetic tree for forensic applications based on whole genome SNPs. Forensic Sci. Int. Genet. 7, 573–580 (2013). 23597787 10.1016/j.fsigen.2013.03.010

[b52] Drummond A. J., Rambaut A., Shapiro B. & Pybus O. G. Bayesian coalescent inference of past population dynamics from molecular sequences. Mol. Biol. Evol. 22, 1185–1192 (2005). 15703244 10.1093/molbev/msi103

[b53] Forster P., Harding R., Torroni A. & Bandelt H.-J. Origin and evolution of Native American mtDNA variation: a reappraisal. Am. J. Hum. Genet. 59, 935–945 (1996). 8808611 PMC1914796

[b54] Saillard J., Forster P., Lynnerup N., Bandelt H.-J. & Nørby S. mtDNA variation among Greenland Eskimos: the edge of the Beringian expansion. Am. J. Hum. Genet. 67, 718–726 (2000). 10924403 10.1086/303038 PMC1287530

[b55] Fenner J. N. Cross-cultural estimation of the human generation interval for use in genetics-based population divergence studies. Am. J. Phys. Anthropol. 128, 415–423 (2005). 15795887 10.1002/ajpa.20188

[b56] Tajima F. Statistical method for testing the neutral mutation hypothesis by DNA polymorphism. Genetics 123, 585–595 (1989). 2513255 10.1093/genetics/123.3.585 PMC1203831

[b57] Fu Y. X. & Li W. H. Statistical tests of neutrality of mutations. Genetics 133, 693–709 (1993). 8454210 10.1093/genetics/133.3.693 PMC1205353

[b58] Excoffier L. & Lischer H. E. Arlequin suite ver 3.5: a new series of programs to perform population genetics analyses under Linux and Windows. Mol. Ecol. Resour. 10, 564–567 (2010). 21565059 10.1111/j.1755-0998.2010.02847.x

[b59] R Core Team. R: A language and environment for statistical computing R Foundation for Statistical Computing, http://www.R-project.org/ (2014).

[b60] Excoffier L., Dupanloup I., Huerta-Sanchez E., Sousa V. C. & Foll M. Robust demographic inference from genomic and SNP data. PLoS Genet. 9, e1003905 (2013). 24204310 10.1371/journal.pgen.1003905 PMC3812088

[b61] Beaumont M. Joint determination of topology, divergence time and immigration in population trees. In: Simulations, genetics and human prehistory eds Matsumura S., Forster P., Renfrew C. McDonald Institute for Archaeological Research (2008).

[b62] Hamilton G., Stoneking M. & Excoffier L. Molecular analysis reveals tighter social regulation of immigration in patrilocal populations than in matrilocal populations. Proc. Natl Acad. Sci. USA 102, 7476–7480 (2005). 15894624 10.1073/pnas.0409253102 PMC1140411

