# Supplementary material for: Large-scale recent expansion of European patrilineages shown by population resequencing
Source: Nat Commun. 2015 May 19;6:7152. doi: 10.1038/ncomms8152 (PMC4441248; doi:10.1038/ncomms8152)
Supplement: Supplementary Information — Supplementary Figures 1-2, Supplementary Tables 1-9 and Supplementary References [file ncomms8152-s1.pdf]

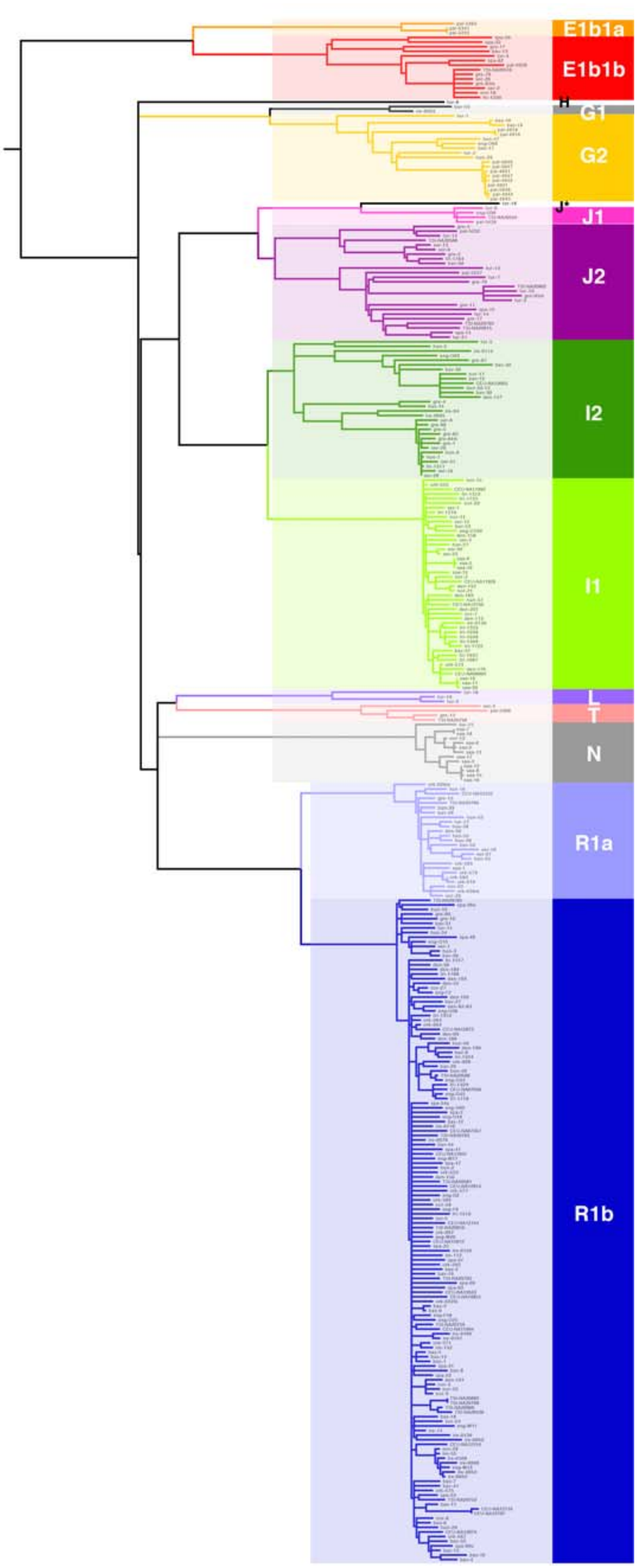

Supplementary Figure 1. Maximum parsimony tree showing sample names. The tree structure is the same as that shown in Figure 1a. Names include a three-letter prefix indicating population, according to the legend of Figure 1.

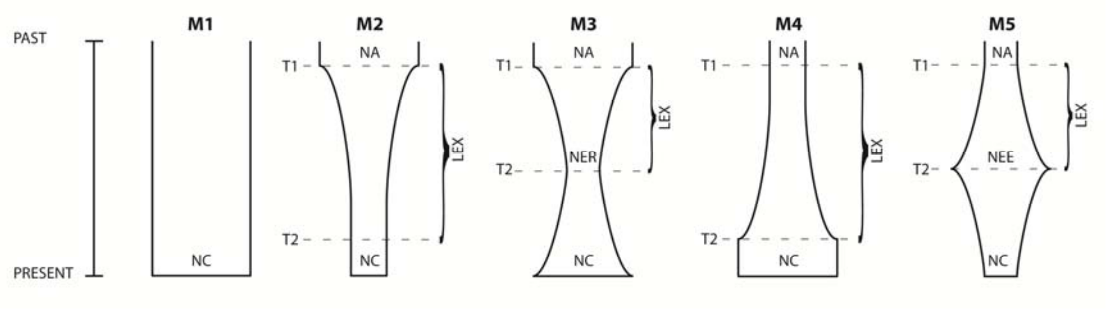

Supplementary Figure 2: Models used in ABC analysis. NC, current effective population size; NA, ancestral effective population size; T1, time of the beginning of the demographic change; T2, time of the end of the demographic change; LEX, length of the expansion/reduction; NER, effective population size after reduction; LEE, effective population size after expansion.

Supplementary Table 1: Sample information.

Sample name	Population	Haplogroup	In final analysis?	Mean raw coverage in final analysed regions (3.72Mb)	No. of singletons (whole dataset)	No. of singletons (within-population)	Total mutations from root (as in Fig. 1)
bas-1	Basque	R1b1b2-S116	yes	43,9	6	6	171
bas-10	Basque	R1b1b2-M153	yes	44,9	11	11	186
bas-11	Basque	R1b1b2-SRY2627	yes	45,8	4	10	174
bas-12	Basque	R1b1b2-S116	yes	53,6	14	14	177
bas-13	Basque	R1b1b2-S116	yes	49,6	5	5	170
bas-14	Basque	I2a-P37.2	yes	45,1	11	119	169
bas-15	Basque	R1b1b2-S116	yes	40,8	4	4	175
bas-16	Basque	G2a-L31	yes	54,7	1	1	197
bas-17	Basque	G2a-L31	yes	57,3	14	45	190
bas-18	Basque	R1b1b2-S116	yes	52,4	10	11	174
bas-19	Basque	G2a-L31	yes	51,6	6	6	202
bas-2	Basque	R1b1b2-S116	yes	36,0	13	13	176
bas-20	Basque	excluded	no, missing data	10,4	n/a	n/a	n/a
bas-3	Basque	R1b1b2-S116	yes	49,7	3	3	171
bas-4	Basque	R1b1b2-M153	yes	41,4	7	7	182
bas-5	Basque	R1b1b2-S116	yes	49,9	4	4	169
bas-6	Basque	R1b1b2-S116	yes	52,9	6	6	171
bas-7	Basque	R1b1b2-S116	yes	47,7	11	11	175
bas-8	Basque	R1b1b2-S116	yes	40,1	14	15	178
bas-9	Basque	R1b1b2-S116	yes	58,9	1	1	169
bav-12	Bavarian	I2b-P214	yes	47,1	11	11	186
bav-13	Bavarian	E1b1b1c-M123	yes	60,5	36	245	196
bav-19	Bavarian	R1b1b2-S116	yes	47,5	10	10	173
bav-26	Bavarian	R1a-M198	yes	54,2	6	7	173
bav-27	Bavarian	R1b1b2-L11	yes	60,8	12	14	176
bav-29	Bavarian	R1b1b2-L11	yes	53,2	6	9	174
bav-30	Bavarian	I2b-P214	yes	48,5	14	14	189
bav-31	Bavarian	R1b1b2-M269	yes	60,5	13	15	172
bav-32	Bavarian	R1b1b2-S116	yes	56,7	8	14	178
bav-38	Bavarian	J2b2-M241	yes	55,3	10	111	177
bav-39	Bavarian	I2b-P214	yes	55,1	18	18	176
bav-40	Bavarian	I2b-P214	yes	47,6	38	39	197
bav-41	Bavarian	R1b1b2-S116	yes	52,1	11	11	175
bav-52	Bavarian	R1a-M198	yes	56,2	11	16	182
bav-53	Bavarian	I1-M253	yes	43,2	7	12	180
bav-54	Bavarian	R1b1b2-S116	yes	57,2	10	10	173

Sample name	Population	Haplogroup	In final analysis?	Mean raw coverage in final analysed regions (3.72Mb)	No. of singletons (whole dataset)	No. of singletons (within-population)	Total mutations from root (as in Fig. 1)
bav-55	Bavarian	G1-M285	yes	51,9	25	131	180
bav-56	Bavarian	R1b1b2-M269	yes	48,9	4	18	175
bav-57	Bavarian	I1-M253	yes	63,0	8	12	180
bav-8	Bavarian	R1b1b2-L11	yes	39,2	6	15	180
CEU-NA06994	French (CEU)	I1-M253	yes	50,9	5	11	180
CEU-NA07048	French (CEU)	R1b1b2-L11	yes	56,2	6	16	178
CEU-NA07357	French (CEU)	R1b1b2-S116	yes	66,8	15	15	178
CEU-NA10851	French (CEU)	R1b1b2-S116	yes	62,2	15	15	178
CEU-NA11829	French (CEU)	I1-M253	yes	57,0	10	16	184
CEU-NA11831	French (CEU)	R1b1b2-S116	yes	49,9	9	9	172
CEU-NA11992	French (CEU)	I1-M253	yes	65,7	11	12	180
CEU-NA11994	French (CEU)	R1b1b2-S116	yes	59,8	10	12	175
CEU-NA12003	French (CEU)	I2b-P214	yes	54,9	14	86	189
CEU-NA12043	French (CEU)	R1b1b2-S116	yes	55,7	13	13	176
CEU-NA12144	French (CEU)	R1b1b2-S116	yes	49,6	14	14	177
CEU-NA12154	French (CEU)	R1b1b2-S116	yes	62,4	13	15	178
CEU-NA12155	French (CEU)	R1a-M198	yes	57,0	13	66	183
CEU-NA12707	French (CEU)	R1b1b2-SRY2627	yes	54,3	0	0	188
CEU-NA12716	French (CEU)	R1b1b2-SRY2627	yes	46,7	3	3	191
CEU-NA12750	French (CEU)	I1-M253	yes	49,0	8	10	179
CEU-NA12812	French (CEU)	R1b1b2-S116	yes	47,1	8	8	171
CEU-NA12814	French (CEU)	R1b1b2-S116	yes	58,9	15	15	178
CEU-NA12872	French (CEU)	R1b1b2-Z18	yes	49,7	10	13	175
CEU-NA12874	French (CEU)	R1b1b2-S116	yes	53,2	10	12	176
den-104	Danish	R1b1b2-L11	yes	57,4	14	15	177
den-113	Danish	I1-M253	yes	46,6	14	14	184
den-150	Danish	R1b1b2-S116	yes	49,1	8	9	172
den-152	Danish	I1-M253	yes	54,2	2	12	181
den-157	Danish	I2b-P214	yes	44,5	16	17	192
den-158	Danish	I1-M253	yes	46,9	12	12	181
den-176	Danish	I1-M253	yes	48,5	10	15	185
den-183	Danish	I1-M253	yes	47,2	10	11	180
den-186	Danish	R1b1b2-Z18	yes	46,0	6	6	173
den-189	Danish	R1b1b2-L11	yes	55,2	12	12	174
den-190	Danish	R1b1b2-L11	yes	46,3	10	21	183
den-191	Danish	R1b1b2-S116	yes	42,3	6	13	176

Sample name	Population	Haplogroup	In final analysis?	Mean raw coverage in final analysed regions (3.72Mb)	No. of singletons (whole dataset)	No. of singletons (within-population)	Total mutations from root (as in Fig. 1)
den-192	Danish	R1b1b2-L11	yes	41,9	14	15	178
den-207	Danish	I1-M253	yes	52,1	12	12	182
den-30	Danish	R1a-M198	yes	49,7	6	59	176
den-34	Danish	R1b1b2-U198	yes	52,9	11	13	175
den-36	Danish	R1b1b2-L11	yes	54,6	9	9	171
den-50-51	Danish	I2b-P214	yes	43,4	10	10	185
den-82-83	Danish	R1b1b2-L11	yes	49,5	8	14	177
den-SN	Danish	R1b1b2-Z18	yes	45,2	8	8	175
eng-F2	English	R1b1b2-U198	yes	53,4	6	10	172
eng-F58	English	R1b1b2-S116	yes	47,3	5	7	171
eng-F6	English	R1b1b2-S116	yes	41,3	10	12	175
eng-G9	English	R1b1b2-S116	yes	40,2	12	12	175
eng-H18	English	R1b1b2-S116	yes	44,6	11	11	175
eng-H38	English	R1b1b2-L11	yes	31,2	5	11	173
eng-N5	English	excluded	no, female	0,2	n/a	n/a	n/a
eng-O109	English	I1-M253	yes	49,5	8	79	182
eng-O15	English	R1b1b2-M269	yes	37,5	6	12	169
eng-O23	English	R1b1b2-S116	yes	43,7	9	9	173
eng-O36	English	J1e-P58	yes	55,0	7	122	189
eng-O44	English	R1b1b2-L11	yes	34,3	3	10	176
eng-O45	English	R1b1b2-L11	yes	40,9	3	10	176
eng-O63	English	I2a-P37.2	yes	50,6	23	71	174
eng-O69	English	G2a-L31	yes	41,2	13	142	190
eng-O93	English	R1b1b2-S116	yes	48,2	11	13	176
eng-W11	English	R1b1b2-S116	yes	39,4	17	18	181
eng-W13	English	R1b1b2-M222	yes	40,2	4	16	179
eng-W17	English	R1b1b2-S116	yes	45,1	10	10	173
eng-W20	English	R1b1b2-S116	yes	42,2	9	9	172
fri-1048	Frisian	I1-M253	yes	51,0	5	5	182
fri-1087	Frisian	I1-M253	yes	59,9	8	8	182
fri-1309	Frisian	I1-M253	yes	60,2	3	3	182
fri-1311	Frisian	I2a-P37.2	yes	57,7	1	65	168
fri-1312	Frisian	I1-M253	yes	49,7	14	14	183
fri-1313	Frisian	R1b1b2-Z18	yes	41,1	8	9	171
fri-1317	Frisian	R1b1b2-L11	yes	50,4	14	14	176
fri-1319	Frisian	I1-M253	yes	48,8	4	5	173
fri-1320	Frisian	E1b1ba2-V13	yes	62,6	11	242	192
fri-1324	Frisian	R1b1b2-L11	yes	47,0	6	15	180

Sample name	Population	Haplogroup	In final analysis?	Mean raw coverage in final analysed regions (3.72Mb)	No. of singletons (whole dataset)	No. of singletons (within-population)	Total mutations from root (as in Fig. 1)
fri-1325	Frisian	I1-M253	yes	42,0	6	7	182
fri-1329	Frisian	R1b1b2-L11	yes	51,5	6	6	178
fri-1516	Frisian	R1b1b2-S116	yes	50,8	15	17	179
fri-1718	Frisian	R1b1b2-L11	yes	53,7	5	6	178
fri-1722	Frisian	I1-M253	yes	46,6	5	5	184
fri-1725	Frisian	I1-M253	yes	61,8	13	13	182
fri-1784	Frisian	J2b2-M241	yes	39,9	10	110	176
fri-1788	Frisian	R1b1b2-L11	yes	43,9	11	12	174
fri-1937	Frisian	I1-M253	yes	48,9	8	8	182
fri-1938	Frisian	I1-M253	yes	47,7	4	5	182
gre-1	Greek	I2a-P37.2	yes	51,0	7	7	175
gre-10	Greek	R1b1b2-M269	yes	55,2	15	15	174
gre-11	Greek	J2a-M410	yes	51,4	36	37	182
gre-12	Greek	T-L131	yes	40,3	10	117	174
gre-15	Greek	R1a-M198	yes	46,3	7	56	173
gre-17	Greek	E1b1b1c-M123	yes	52,7	35	66	195
gre-2	Greek	J2b2-M241	yes	47,4	16	44	177
gre-3	Greek	J2b1-M205	yes	43,7	17	48	181
gre-4	Greek	I2a-P37.2	yes	49,2	12	53	171
gre-5	Greek	I2a-P37.2	yes	41,5	5	5	171
gre-77	Greek	J2a2-M67	yes	51,9	34	40	185
gre-78	Greek	E1b1ba2-V13	yes	48,2	8	8	189
gre-79	Greek	J2a-M410	yes	51,1	40	41	187
gre-80	Greek	R1b1b2-M269	yes	56,8	13	13	172
gre-82	Greek	I2a-P37.2	yes	42,0	8	8	175
gre-83m	Greek	E1b1ba2-V13	yes	61,1	8	8	189
gre-84m	Greek	I2a-P37.2	yes	52,2	6	6	173
gre-85m	Greek	J2a-M410	yes	51,4	16	63	209
gre-86	Greek	I2a-P37.2	yes	53,2	5	5	170
gre-87	Greek	I2b-P214	yes	43,3	31	73	187
hun-1	Hungarian	I2a-P37.2	yes	44,2	1	1	168
hun-2	Hungarian	R1b1b2-S116	yes	51,2	11	11	174
hun-25	Hungarian	R1a-M198	yes	61,0	4	18	188
hun-26	Hungarian	R1b1b2-S116	yes	58,7	9	11	174
hun-27	Hungarian	I1-M253	yes	57,7	10	11	179
hun-28	Hungarian	R1a-M198	yes	59,4	5	5	180
hun-29	Hungarian	G2a-L31	yes	49,4	30	34	189
hun-3	Hungarian	R1b1b2-M269	yes	63,7	3	16	175

Sample name	Population	Haplogroup	In final analysis?	Mean raw coverage in final analysed regions (3.72Mb)	No. of singletons (whole dataset)	No. of singletons (within-population)	Total mutations from root (as in Fig. 1)
hun-33	Hungarian	R1b1b2-M269	yes	52,6	11	13	170
hun-34	Hungarian	R1b1b2-M269	yes	47,8	10	11	170
hun-35	Hungarian	R1a-M198	yes	59,7	4	4	179
hun-37	Hungarian	I1-M253	yes	68,4	15	17	185
hun-38	Hungarian	R1a-M198	yes	69,7	4	12	179
hun-39	Hungarian	R1a-M198	yes	44,6	6	7	173
hun-40	Hungarian	R1b1b2-L11	yes	56,2	7	13	178
hun-43	Hungarian	R1a-M198	yes	70,4	17	18	185
hun-46	Hungarian	R1b1b2-L11	yes	58,7	9	14	179
hun-47	Hungarian	G2a-L31	yes	66,8	18	37	192
hun-5	Hungarian	I2-P125	yes	48,0	40	57	171
hun-9	Hungarian	I2a-P37.2	yes	49,4	9	9	176
ire-0052	Irish	R1b1b2-M222	yes	51,3	1	1	177
ire-0053	Irish	R1b1b2-M222	yes	66,1	5	5	181
ire-0059	Irish	R1b1b2-S116	yes	70,0	19	19	184
ire-0068	Irish	R1b1b2-M222	yes	46,9	7	7	182
ire-0078	Irish	R1b1b2-S116	yes	32,4	6	6	169
ire-0093	Irish	G1-M285	yes	60,3	10	116	164
ire-0095	Irish	I2a-P37.2	yes	43,0	19	19	168
ire-0100	Irish	R1b1b2-M222	yes	55,0	2	3	177
ire-0107	Irish	R1b1b2-S116	yes	55,0	2	3	175
ire-0109	Irish	R1b1b2-S116	yes	37,5	7	7	179
ire-0114	Irish	I2b-P214	yes	52,5	56	74	188
ire-0116	Irish	R1b1b2-S116	yes	43,7	9	9	172
ire-0120	Irish	R1b1b2-S116	yes	61,3	16	16	179
ire-0130	Irish	I1-M253	yes	49,8	9	82	185
ire-0136	Irish	R1b1b2-S116	yes	54,1	14	14	179
ire-112	Irish	R1b1b2-S116	yes	73,8	13	13	176
ire-132	Irish	R1b1b2-S116	yes	80,4	4	9	172
ire-55	Irish	R1b1b2-M222	yes	59,6	2	2	175
ire-74	Irish	R1b1b2-S116	yes	52,7	5	5	169
ire-94	Irish	I2a-P37.2	yes	53,4	26	27	176
nor-13	Norwegian	N1c1-M178	yes	54,9	1	120	178
nor-14	Norwegian	I1-M253	yes	55,9	9	10	178
nor-15	Norwegian	I1-M253	yes	55,0	17	17	185
nor-16	Norwegian	R1a-M198	yes	64,8	9	13	178
nor-17	Norwegian	I2b-P214	yes	47,5	11	83	186
nor-18	Norwegian	E1b1ba2-V13	yes	45,7	10	241	191

Sample name	Population	Haplogroup	In final analysis?	Mean raw coverage in final analysed regions (3.72Mb)	No. of singletons (whole dataset)	No. of singletons (within-population)	Total mutations from root (as in Fig. 1)
nor-2	Norwegian	I1-M253	yes	57,1	7	7	180
nor-20	Norwegian	I1-M253	yes	48,6	15	16	184
nor-21	Norwegian	I1-M253	yes	56,5	2	8	181
nor-23	Norwegian	R1a-M198	yes	43,0	6	6	177
nor-24	Norwegian	R1b1b2-S116	yes	48,9	12	13	176
nor-25	Norwegian	R1b1b2-S116	yes	51,3	6	6	175
nor-26	Norwegian	R1a-M198	yes	66,3	5	5	176
nor-27	Norwegian	R1b1b2-U198	yes	61,1	4	8	170
nor-28	Norwegian	R1b1b2-S116	yes	55,1	9	9	172
nor-29	Norwegian	R1b1b2-M222	yes	50,8	2	12	175
nor-3	Norwegian	R1b1b2-S116	yes	58,8	4	4	173
nor-7	Norwegian	I1-M253	yes	56,6	14	16	184
nor-8	Norwegian	R1b1b2-S116	yes	38,7	7	9	172
nor-9	Norwegian	R1b1b2-S116	yes	46,6	3	3	172
ork-004	Orcadian	R1b1b2-Z18	yes	42,0	2	4	167
ork-007	Orcadian	R1b1b2-S116	yes	50,8	9	9	172
ork-008	Orcadian	R1b1b2-L11	yes	45,3	11	17	179
ork-010	Orcadian	R1a-M198	yes	33,0	3	3	181
ork-022m	Orcadian	R1b1b2-S116	yes	52,6	9	9	172
ork-026m	Orcadian	R1a-M198	yes	43,4	12	13	169
ork-036m	Orcadian	R1a-M198	yes	50,2	9	13	181
ork-525	Orcadian	I1-M253	yes	33,5	2	2	170
ork-552	Orcadian	R1b1b2-S116	yes	35,3	11	11	174
ork-564	Orcadian	R1b1b2-Z18	yes	42,7	4	4	167
ork-565	Orcadian	R1a-M198	yes	38,5	12	12	180
ork-567	Orcadian	R1b1b2-S116	yes	46,9	7	13	177
ork-571	Orcadian	R1b1b2-S116	yes	49,1	4	8	171
ork-573	Orcadian	I1-M253	yes	46,3	1	8	176
ork-575	Orcadian	R1b1b2-S116	yes	57,5	8	8	172
ork-577	Orcadian	R1b1b2-S116	yes	44,9	15	15	178
ork-579	Orcadian	R1a-M198	yes	44,7	6	6	182
ork-583	Orcadian	R1a-M198	yes	44,7	0	0	178
ork-584	Orcadian	R1b1b2-S116	yes	50,6	8	8	171
ork-585	Orcadian	R1b1b2-S116	yes	32,6	12	12	175
pal-4918	Palestinian	G2a-L31	yes	41,8	0	0	198
pal-4919	Palestinian	G2a-L31	yes	51,3	1	1	199
pal-4921	Palestinian	G2a-L31	yes	48,9	0	0	194
pal-4922	Palestinian	G2a-L31	yes	35,6	2	2	196

Sample name	Population	Haplogroup	In final analysis?	Mean raw coverage in final analysed regions (3.72Mb)	No. of singletons (whole dataset)	No. of singletons (within-population)	Total mutations from root (as in Fig. 1)
pal-4929	Palestinian	E1b1ba3-V22	yes	53,9	22	130	202
pal-4940	Palestinian	G2a-L31	yes	55,4	3	3	196
pal-4941	Palestinian	G2a-L31	yes	42,4	1	1	195
pal-4942	Palestinian	G2a-L31	yes	70,6	1	1	196
pal-4943	Palestinian	G2a-L31	yes	59,0	0	0	195
pal-4947	Palestinian	G2a-L31	yes	68,0	2	2	196
pal-5047	Palestinian	G2a-L31	yes	48,2	3	3	196
pal-5048	Palestinian	G2a-L31	yes	52,7	0	0	195
pal-5225	Palestinian	E1b1a1-M58	yes	59,8	0	0	178
pal-5232	Palestinian	J2b1-M205	yes	50,5	17	76	182
pal-5257	Palestinian	J2a-M410	yes	51,1	39	77	183
pal-5341	Palestinian	E1b1a1-M58	yes	45,6	0	0	178
pal-5365	Palestinian	E1b1a8a-U175	yes	85,2	21	22	181
pal-5366	Palestinian	T-P322	yes	57,4	41	145	195
pal-5370	Palestinian	excluded	no, missing data	3,6	n/a	n/a	n/a
pal-5420	Palestinian	J1e-P58	yes	51,6	6	90	189
saa-1	Saami	R1a-M198	yes	48,8	11	121	179
saa-10	Saami	I1-M253	yes	55,5	0	0	181
saa-11	Saami	I1-M253	yes	63,8	1	1	183
saa-12	Saami	N1c1-M178	yes	63,7	0	0	184
saa-13	Saami	N1c1-M178	yes	52,8	4	4	185
saa-14	Saami	I1-M253	yes	53,3	9	10	179
saa-15	Saami	N1c1-M178	yes	46,7	1	1	185
saa-16	Saami	N1c1-M178	yes	50,8	0	0	184
saa-17	Saami	N1c1-M178	yes	68,0	6	6	181
saa-18	Saami	N1c1-M178	yes	71,5	0	0	181
saa-19	Saami	I1-M253	yes	43,8	0	0	182
saa-2	Saami	N1c1-M178	yes	52,1	1	1	182
saa-20	Saami	I1-M253	yes	32,2	1	1	183
saa-3	Saami	N1c1-M178	yes	53,6	5	5	183
saa-4	Saami	excluded	no, missing data	0,9	n/a	n/a	n/a
saa-5	Saami	I1-M253	yes	39,4	1	1	182
saa-6	Saami	N1c1-M178	yes	56,8	4	4	185
saa-7	Saami	N1c1-M178	yes	54,7	0	0	181
saa-8	Saami	N1c1-M178	yes	49,7	1	1	185
saa-9	Saami	I1-M253	yes	55,4	0	0	181
ser-1	Serbian	I1-M253	yes	42,5	8	8	177
ser-12	Serbian	I1-M253	yes	45,8	10	11	180

Sample name	Population	Haplogroup	In final analysis?	Mean raw coverage in final analysed regions (3.72Mb)	No. of singletons (whole dataset)	No. of singletons (within-population)	Total mutations from root (as in Fig. 1)
ser-13	Serbian	J2b2-M241	yes	41,3	6	6	171
ser-16	Serbian	I2a-P37.2	yes	44,0	6	6	173
ser-19	Serbian	R1a-M198	yes	47,3	10	11	191
ser-2	Serbian	E1b1ba2-V13	yes	47,6	13	13	194
ser-20	Serbian	I2a-P37.2	yes	52,1	3	4	170
ser-21	Serbian	I2a-P37.2	yes	44,2	7	7	174
ser-23	Serbian	I1-M253	yes	55,3	1	1	175
ser-24	Serbian	excluded	no, missing data	68,4	n/a	n/a	n/a
ser-26	Serbian	E1b1ba2-V13	yes	57,3	8	8	189
ser-27	Serbian	R1a-M198	yes	45,4	5	9	189
ser-28	Serbian	I2a-P37.2	yes	60,9	0	0	167
ser-3	Serbian	T-L208	yes	42,5	49	135	192
ser-30	Serbian	I1-M253	yes	61,8	3	3	177
ser-4	Serbian	I1-M253	yes	44,7	13	13	182
ser-5	Serbian	R1b1b2-S116	yes	40,7	9	15	172
ser-6	Serbian	I2a-P37.2	yes	48,0	9	9	174
ser-7	Serbian	R1b1b2-M269	yes	48,5	10	16	173
ser-9	Serbian	J2b2-M241	yes	49,0	8	8	173
spa-14	Spanish	J2a2-M67	yes	60,4	9	34	180
spa-15	Spanish	J2a-M410	yes	60,0	43	44	190
spa-2	Spanish	R1b1b2-S116	yes	53,3	15	15	178
spa-20	Spanish	E1b1b-M215	yes	50,2	34	35	197
spa-22	Spanish	R1b1b2-S116	yes	64,9	8	8	172
spa-25	Spanish	R1b1b2-S116	yes	50,7	8	8	171
spa-26u	Spanish	R1b1b2-M269	yes	43,4	22	24	181
spa-31	Spanish	R1b1b2-S116	yes	51,1	9	9	173
spa-32	Spanish	E1b1b1b-M81	yes	57,0	31	31	193
spa-37	Spanish	R1b1b2-S116	yes	58,7	14	14	177
spa-40	Spanish	R1b1b2-M269	yes	51,8	20	25	182
spa-41	Spanish	R1b1b2-S116	yes	49,5	13	13	176
spa-47	Spanish	R1b1b2-S116	yes	56,4	13	13	176
spa-51	Spanish	excluded	no, female	0,3	n/a	n/a	n/a
spa-53	Spanish	R1b1b2-S116	yes	43,7	8	10	174
spa-54u	Spanish	R1b1b2-S116	yes	46,9	6	7	170
spa-60	Spanish	R1b1b2-S116	yes	42,9	19	19	182
spa-60u	Spanish	R1b1b2-S116	yes	55,5	9	16	180
spa-62	Spanish	E1b1ba3-V22	yes	56,6	15	66	194
spa-63	Spanish	R1b1b2-S116	yes	47,6	14	14	177

Sample name	Population	Haplogroup	In final analysis?	Mean raw coverage in final analysed regions (3.72Mb)	No. of singletons (whole dataset)	No. of singletons (within-population)	Total mutations from root (as in Fig. 1)
TSI-NA20510	Italian (TSI)	E1b1ba2-V13	yes	50,9	11	241	192
TSI-NA20538	Italian (TSI)	R1b1b2-S116	yes	53,3	5	5	180
TSI-NA20543	Italian (TSI)	J1e-P58	yes	58,8	12	95	194
TSI-NA20581	Italian (TSI)	R1b1b2-S116	yes	55,3	12	12	175
TSI-NA20586	Italian (TSI)	R1b1b2-L11	yes	59,7	1	12	174
TSI-NA20588	Italian (TSI)	J2b-M102	yes	54,9	34	63	169
TSI-NA20752	Italian (TSI)	R1b1b2-S116	yes	52,9	11	14	177
TSI-NA20758	Italian (TSI)	T-L131	yes	50,3	9	116	173
TSI-NA20759	Italian (TSI)	R1b1b2-S116	yes	46,7	7	9	172
TSI-NA20765	Italian (TSI)	J2a2-M67	yes	52,7	23	23	184
TSI-NA20783	Italian (TSI)	R1b1b2-S116	yes	58,5	11	11	174
TSI-NA20785	Italian (TSI)	R1b1b2-M269	yes	48,9	12	14	171
TSI-NA20792	Italian (TSI)	R1b1b2-S116	yes	51,0	12	12	175
TSI-NA20796	Italian (TSI)	R1a-M198	yes	46,0	12	61	178
TSI-NA20798	Italian (TSI)	R1b1b2-S116	yes	46,4	0	0	178
TSI-NA20803	Italian (TSI)	R1b1b2-S116	yes	54,4	0	0	178
TSI-NA20805	Italian (TSI)	J2a-M410	yes	36,6	13	62	206
TSI-NA20806	Italian (TSI)	R1b1b2-S116	yes	42,2	2	2	176
TSI-NA20810	Italian (TSI)	R1b1b2-S116	yes	47,1	9	9	172
TSI-NA20815	Italian (TSI)	J2a2-M67	yes	50,9	22	22	183
tur-1	Turkish	G2a-L31	yes	38,7	49	49	181
tur-10	Turkish	J2a-M410	yes	44,1	14	14	207
tur-11	Turkish	R1b1b2-M269	yes	50,4	14	56	173
tur-12	Turkish	J2b1-M205	yes	44,2	10	69	175
tur-13	Turkish	J2a-M410	yes	46,9	59	60	193
tur-14	Turkish	J2a2-M67	yes	53,3	37	37	188
tur-15	Turkish	N1c1-M178	yes	38,5	17	124	182
tur-16	Turkish	J-M304	yes	54,8	58	58	200
tur-17	Turkish	R1a-M198	yes	50,0	5	63	180
tur-18	Turkish	L3-M357	yes	56,8	53	54	184
tur-19	Turkish	L-M11	yes	45,3	6	6	173
tur-2	Turkish	G2a-L31	yes	50,1	25	51	184
tur-21	Turkish	J2a2-M67	yes	44,9	8	28	179
tur-3	Turkish	J2a-M410	yes	56,1	12	12	205
tur-4	Turkish	E1b1b-M215	yes	52,6	39	247	198
tur-5	Turkish	I2-P125	yes	57,1	60	125	191
tur-6	Turkish	J1e-P58	yes	56,4	12	51	193
tur-7	Turkish	J2a-M410	yes	52,6	46	49	194

Sample name	Population	Haplogroup	In final analysis?	Mean raw coverage in final analysed regions (3.72Mb)	No. of singletons (whole dataset)	No. of singletons (within-population)	Total mutations from root (as in Fig. 1)
tur-8	Turkish	H1-M52	yes	53,7	128	128	177
tur-9	Turkish	L-M11	yes	53,6	10	10	177

Supplementary Table 2. Variant validation summary.

Comparison	No. of overlapping samples	No. of overlapping sites	No. of calls compared	No of. FP/FN calls (%)	No. of sites with false calls
Our data vs CompleteGenomics	4	888	3542	0 (0%)/ 0 (0%)	0
Our data vs OMNI 2.5	39	241	6916	9 (0.13%)/ 126 (1.82%)	19
CompleteGenomics vs OMNI 2.5	49	263	5258	150 (2.85%)/ 124 (2.36)	30

Supplementary Table 3. Correlation among latitude, longitude and diversity measures

		All 17 populations		13 populations showing recent expansion BSPs	
**Comparison**	**Test**	**R**	**p-value**	**R**	**p-value**
median no. of singletons vs latitude	Pearson	-0,407	0,105	-0,311	0,302
	Spearman	-0,393	0,118	-0,299	0,321
median no. of singletons vs longitude	Pearson	0,349	0,17	0,107	0,727
	Spearman	0,034	0,898	0,065	0,833
sd of no. of singletons vs latitude	Pearson	-0,2	0,441	0,016	0,959
	Spearman	-0,216	0,405	-0,132	0,667
sd of no. of singletons vs longitude	Pearson	0,319	0,212	0,206	0,499
	Spearman	0,296	0,25	0,261	0,388
S vs latitude	Pearson	-0,627	**0,007**	-0,351	0,239
	Spearman	-0,713	**0,001**	-0,413	0,161
S vs longitude	Pearson	0,601	**0,011**	0,583	**0,036**
	Spearman	0,529	**0,029**	0,564	**0,045**
Age of minimum Ne before recent expansion vs latitude	Pearson	nd	nd	0,035	0,909
	Spearman	nd	nd	-0,063	0,837
Age of minimum Ne before recent expansion vs longitude	Pearson	nd	nd	-0,179	0,558
	Spearman	nd	nd	-0,044	0,887

nd: not determined

Supplementary Table 4. Times at which effective population sizes were at a minimum prior to expansion.

**Population**	**Time (95% CI)/Y**
bas	3023 (2773-3285)
bav	3424 (3141-3719)
CEU	3825 (3510-4160)
den	3639 (3337-4029)
eng	3245 (2979-3529)
fri	3452 (3168-3752)
hun	3728 (3422-4054)
ire	3471 (3183-3774)
nor	3280 (3008-3563)
ork	2862 (2687-3110)
ser	2287 (2098-2486)
spa	3630 (3331-3945)
TSI	3293 (3021-3579)

Population abbreviations are as in Figure 1. CI: confidence interval; Y: years.

Supplementary Table 5. ABC proportion of true and false positives based on 1,000 PODS simulated under each model**.**

Simulated model	True postitives	False positives
M1	0.744	0.062
M2	0.581	0.089
M3	0.481	0.114
M4	0.738	0.074
M5	0.701	0.101
M2+M3	0.796	0.047
M4+M5	0.937	0.044

The constant population model (M1) has a proportion of true positives over 70%, while for the other models the proportion of true positives ranges from 48% (M3) to 74% (M4). This may be due to the high similarity between M2 and M3 and between M4 and M5, making it difficult to distinguish between these pairs of models. For this reason, we also calculate the proportion of true and false positives combining models sharing the same ancient demographic event i.e. reduction (M2+M3) or expansion (M4+M5), reaching a high proportion of true positives, of 80% and 94% respectively. To estimate posterior probabilities (Supplementary Table 6) we use M1 plus the combined models.

Supplementary Table 6. Posterior probabilities of the models computed for each population based on the multinomial logistic regression approach with 50,000 retained simulations.

Population	Models			Skyline
	M1	M2+M3	M4+M5	
bas	0.05	**0.75**	0.20	
bav	0.15	0.12	**0.73**	*
CEU	0.04	0.57	0.49	
den	0.06	0.56	0.38	
eng	0.01	0.06	**0.93**	*
fri	0.04	**0.75**	0.21	
gre	0.14	0.13	**0.73**	*
hun	0.10	0.30	0.60	
ire	0.02	0.54	0.44	
nor	0.07	0.64	0.29	
ork	0.08	0.56	0.36	
pal	0.11	0.31	0.58	
saa	0.03	**0.85**	0.12	*
ser	0.18	0.50	0.32	
spa	0.09	0.18	**0.73**	*
TSI	0.05	0.39	0.56	
tur	0.14	0.06	**0.80**	*

Bold text indicates models with high support (P>0.7). The “Skyline” column indicates the presence of agreement (*) between the ABC confidently selected model and the skyline reconstruction.

In all 17 populations, M1 is never selected as the best model, with posterior probabilities ranging from 1% (eng) to 18% (ser). In most cases in which we obtain a clear support (above 70%, shown in bold) for the reduction (M2+M3) or the expansion (M4+M5) models, the ABC results agree with the Bayesian Skyline analysis. For some populations the posterior probability of the selected model is very high (93% for eng or 85% for saa). In some cases we are not able to confidently assign a support to a specific demography, with highest probabilities of about 50%.

Supplementary Table 7. Estimated parameters from Approximate Bayesian Computation for those populations showing a clear support for a specific demography.

Population	Model	Parameter	Median	2.5% CI	97.5% CI	R^2
saa	M2	NA	4661	1169	17403	0.29
		NC	269	64	870	0.15
		T2	510	30	877	0.00*
		T1	12475	1841	19265	0.13
bas	M2	NA	3398	1134	15556	0.29
		NC	388	87	955	0.14
		T2	486	30	874	0.00*
		T1	12332	1584	19256	0.11
eng	M4	NA	681	294	983	0.41
		NC	13961	4834	19708	0.22
		T2	493	30	875	0.00*
		T1	8247	3196	17677	0.45
spa	M4	NA	826	468	991	0.41
		NC	7609	2134	19038	0.21
		T2	413	30	867	0.00*
		T1	8032	1936	18475	0.45
bav	M4	NA	870	566	995	0.41
		NC	9483	3257	19244	0.23
		T2	421	30	865	0.00*
		T1	13343	5749	18996	0.47
fri	M2	NA	3787	1241	15593	0.29
		NC	530	125	973	0.15
		T2	437	30	871	0.00*
		T1	11399	1430	19165	0.13
gre	M4	NA	860	552	993	0.42
		NC	12958	4934	19593	0.24
		T2	468	30	871	0.00*
		T1	14617	6716	19158	0.47
tur	M4	NA	883	575	995	0.39
		NC	17150	10231	19880	0.24
		T2	504	30	875	0.00*
		T1	17568	11758	19599	0.43

Populations listed are those for which a model was chosen (Table S6). A point estimate (median) and the 95% credible interval (CI) are given. Times are in years and population sizes are in number of individuals (haploid locus). R^2 is the proportion of the parameter variance explained by the summary statistics.
* Parameters with a $R^2 < 0.1$ are considered poorly estimated.

Within the M2+M3 and M4+M5 sets, we estimate parameters of the less parameterised model, namely M2 and M4. The exponential reduction model M2 is favoured for saa, bas and fri, for which we estimate ancestral effective population sizes of about 4000-5000 reducing to about 300-500 currently. For eng, spa, bav, gre and tur the most supported model is M4, with an exponential growth starting with about 700-900 individuals and reaching current effective population sizes of 8000 - 17,000 depending on the population.

Supplementary Table 8. MSY-haplogroups in ancient European samples.

culture	country	YBP[a]	hg	simple hg[a]	N	Ref
Mesolithic HG	Luxembourg	8000	I2a1b-L178	I2	1	10
Early Neolithic	Hungary	7700	I2a-L460	I2	1	7
Hunter Gatherer	Sweden	7700	I2a1-P37.2	I2	1	4
Hunter Gatherer	Sweden	7700	I2a1a1a-L672	I2	1	4
Hunter Gatherer	Sweden	7700	I2a1b-M423	I2	1	4
Hunter Gatherer	Sweden	7700	I2a1b2a1-L147.2	I2	1	4
Hunter Gatherer	Sweden	7700	I2c2-PF3827	I2	5	4
Early Neolithic	Hungary	7600	H2-L281	H2	1	4
Hunter Gatherer	Russia	7600	R1b1-L278	R1b1	1	4
Hunter Gatherer	Russia	7300	R1a1-M459	R1a	1	4
Early Neolithic	Spain	7300	F*-P135	F*	1	4
Early Neolithic	Spain	7300	I2a1b1-L161.1	I2	1	4
Early Neolithic	Germany	7200	T1a-PF5604	T	1	4
Early Neolithic	Germany	7100	G2a2a-PF3147	G2a	1	4
Early Neolithic	Germany	7100	G2a2a-PF3185	G2a	1	4
Early Neolithic	Germany	7100	G2a2a1-PF3170	G2a	1	4
Early Neolithic	Hungary	7100	C1a2-V20/V184	C1	2	7
Late Bronze Age	Hungary	7100	J2a1-S57	J2	1	7
Early Neolithic	Spain	7100	R1b1-M415	R1b1	1	4
Early Neolithic	Germany	7000	G2a2a1-PF3155	G2a	1	4
Early Neolithic	Spain	7000	E1b1b1a1b1a-V13	E1b1b	1	12
Early Neolithic	Spain	7000	G2a-P15	G2a	5	12
Early Neolithic	Spain	6900	C1a2-V20	C1	1	11
Early Neolithic	Hungary	6400	I2a-L460	I2	1	7
Early Neolithic	Germany	6300	F-M89	F*	2	3
Early Neolithic	Germany	6200	G2a2b-S126	G2a	1	3
Middle Neolithic	Germany	5900	F*-P316	F*	1	4
Middle Neolithic	Spain	5800	H2/I2a1a1-L672	I2	1	4
Middle Neolithic	Spain	5800	I2a2a1-CTS9183	I2	1	4
Middle Neolithic	Germany	5600	R*?-P224	R*	1	4
Early Neolithic	Hungary	5600	F-M89	F*	3	8
Early Neolithic	Hungary	5600	G2a-P15	G2a	5	8
Early Neolithic	Hungary	5600	G2a2b-S126	G2a	4	8
Early Neolithic	Hungary	5600[b]	I-M170	I*	1	8
Early Neolithic	Hungary	5600[b]	I1-M253	I1	1	8
Early Neolithic	Hungary	5600[b]	I2a1-P37.2	I2	1	8
Neolithic HG	Sweden	5600	I2a1-P37.2	I2	1	13
Middle Neolithic	Germany	5200	I2a1b1a-L1498	I2	1	4
Middle Neolithic	Italy	5200	G2a2a1b-L91	G2a	1	9
Steppe Yamnaya	Russia	5100	R1b1a-P297	R1b1a	1	4
Steppe Yamnaya	Russia	5100	R1b1a2a-L49.1	R1b1a	1	4
Steppe Yamnaya	Russia	5100	R1b1a2a2-CTS1078	R1b1a	1	4
Steppe Yamnaya	Russia	5100	R1b1a2a2-CTS1078	R1b1a	1	4
Steppe Yamnaya	Russia	5100	R1b1a2a2-Z2105	R1b1a	1	4
Middle Neolithic	France	5000	G2a-P15	G2a	20	1
Middle Neolithic	France	5000	I2a1-P37.2	I2	2	1
Steppe Yamnaya	Russia	4900	R1b1a2a2-CTS1078	R1b1a	1	4
Steppe Yamnaya	Russia	4800	R1b1a2a2-Z2105	R1b1a	1	4
Middle Neolithic	France	4700	I2a1	I2	2	2
Late Neolithic	Germany	4600	R1b-M343	R1b	1	6
Late Neolithic	Germany	4600	R1b1a2-M269	R1b1a	1	6
Late Neolithic	Germany	4400	R1a1a1-M417	R1a	1	4
Late Neolithic	Germany	4300	R1b1a2a1a2-P312	R1b1a	1	4
Early Bronze Age	Germany	4100	I2-L68	I2	1	4
Early Bronze Age	Germany	4100	I2a2-L368	I2	1	4
Early Bronze Age	Germany	4100	I2c2-PF3827	I2	1	4
Late Neolithic	Germany	4100	R1a1-SRY10831.2	R1a	1	5
Early Bronze Age	Germany	3100	R1a1a1b1a2-S204	R1a	1	4

YBP: years before present; hg: haplogroup; N: number of samples; HG: hunter-gatherer
[a] As used for Figure 3.
[b] Assumed from archaeological context.

Supplementary Table 9. Software tools and parameters used in data analysis.

Function	Tool	Package	Parameters
Call bases	Illumina Bustard		
Quality control	FastQC		
Map reads	Stampy v1.0.20		-g GRCh37 -h GRCh37 --fast --insertsize=330 --insertsd=120 –xa-max=2 --xa-max-discordant=2
Local realignment	RealignerTargetCreator	GATK v2.6-5	-R GRCh37
	IndelRealigner		-R GRCh37 -targetIntervals file_made_by_RealignerTargetCreator
Duplicate marking	MarkDuplicates	Picard v1.86	N/A
Base quality score recalibration	BaseRecalibrator	GATK v2.6-5	-R GRCh37 --covariate CycleCovariate --covariate ContextCovariate -knownSites broad_dbsnp -knownSites known_phase1_indels -knownSites known_mills_indels<br>(plus ReadGroupCovariate and QualityScoreCovariate by default)
Raw sequence depth	CalculateHsMetrics	Picard v1.93	BAIT_INTERVALS= analysed_coordinate_file TARGET_INTERVALS=original_8target_intervals
SNP calling	mpileup	SAMtools v0.1.19	-Q 20 -q 50 -I -D -S -g -O -s -f B37 -l analysed_coordinate_file
	bcftools view		-v -c -g
Filtering	Vcftools v0.1.11 and Perl scripts		Maximum 3x average DP per site across all samples<br>SnpCluster [2,4]<br>Strand bias (SP>13)<br>Minimum DP 6<br>Remove heterozygous calls<br>Maximum missing data per site – 5%<br>Maximum missing data per sample – 5%

Supplementary References

1. Lacan M*, et al.* Ancient DNA reveals male diffusion through the Neolithic Mediterranean route. *Proc Natl Acad Sci U S A* **108**, 9788-9791 (2011).

2. Lacan M. La Néolithisation du bassin méditerranéen: Apports de l'ADN ancien. (ed^(eds). Université Toulouse III Paul Sabatier (2011).

3. Haak W*, et al.* Ancient DNA from European early neolithic farmers reveals their near eastern affinities. *PLoS Biol* **8**, e1000536 (2010).

4. Haak W*, et al.* Massive migration from the steppe is a source for Indo-European languages in Europe. *BIoArxiv*, (2015).

5. Haak W*, et al.* Ancient DNA, Strontium isotopes, and osteological analyses shed light on social and kinship organization of the Later Stone Age. *Proc Natl Acad Sci U S A* **105**, 18226-18231 (2008).

6. Lee EJ*, et al.* Emerging genetic patterns of the European Neolithic: perspectives from a late Neolithic Bell Beaker burial site in Germany. *Am J Phys Anthropol* **148**, 571-579 (2012).

7. Gamba C*, et al.* Genome flux and stasis in a five millennium transect of European prehistory. *Nat Commun* **5**, 5257 (2014).

8. Szécsényi-Nagy A*, et al.* Tracing the genetic origin of Europe's first farmers reveals insights into their social organization. *BIoArxiv*, (2014).

9. Keller A*, et al.* New insights into the Tyrolean Iceman's origin and phenotype as inferred by whole-genome sequencing. *Nat Commun* **3**, 698 (2012).

10. Lazaridis I*, et al.* Ancient human genomes suggest three ancestral populations for present-day Europeans. *Nature* **513**, 409-413 (2014).

11. Olalde I*, et al.* Derived immune and ancestral pigmentation alleles in a 7,000-year-old Mesolithic European. *Nature* **507**, 225-228 (2014).

12. Lacan M*, et al.* Ancient DNA suggests the leading role played by men in the Neolithic dissemination. *Proc Natl Acad Sci U S A* **108**, 18255-18259 (2011).

13. Skoglund P*, et al.* Genomic diversity and admixture differs for Stone-Age Scandinavian foragers and farmers. *Science* **344**, 747-750 (2014).
